# Sex-Specific Mediating Role of Insulin Resistance and Inflammation in the Effect of Adiposity on Blood Pressure of Prepubertal Children

**DOI:** 10.1371/journal.pone.0132097

**Published:** 2015-06-30

**Authors:** Liane Correia-Costa, Ana Cristina Santos, Milton Severo, António Guerra, Franz Schaefer, Alberto Caldas Afonso, Henrique Barros, Ana Azevedo

**Affiliations:** 1 EPIUnit—Institute of Public Health, University of Porto, Porto, Portugal; 2 Division of Pediatric Nephrology, Integrated Pediatric Hospital, Centro Hospitalar São João, Porto, Portugal; 3 Department of Clinical Epidemiology, Predictive Medicine and Public Health, University of Porto Medical School, Porto, Portugal; 4 Division of Pediatric Nutrition, Integrated Pediatric Hospital, Centro Hospitalar São João, Porto, Portugal; 5 Division of Pediatric Nephrology, Center for Pediatrics and Adolescent Medicine, University of Heidelberg, Heidelberg, Germany; Medical University Innsbruck, AUSTRIA

## Abstract

**Objective:**

To evaluate the association between obesity indices and blood pressure (BP) at 4 years of age, in each sex, and to quantify to which extent this association is mediated by inflammation and insulin resistance (IR).

**Materials and Methods:**

We studied 1250 4-year-old children selected from the population-based birth cohort Generation XXI. Associations between body mass index (BMI) z-score and waist-to-height ratio (WHtR), office BP, inflammation (high sensitivity C-reactive protein) and IR (HOMA-IR index) were assessed. Path Analysis, a modified multivariate regression approach, was applied to test causal models and quantify direct and indirect effects of predictors of systolic (SBP) and diastolic BP (DBP).

**Results:**

SBP and DBP increased significantly with BMI and WHtR in both sexes. There was a strong direct association (explaining 74.1-93.2% of the total association) of both measures of adiposity with SBP, in both sexes. This association was additionally indirectly mediated by IR, particularly regarding WHtR (20.5% in girls and 9.4% in boys). Mediation by inflammation did not reach statistical significance in either sex. Regarding DBP, the direct effect of adiposity was strong (>95% for BMI and WHtR in boys) and the mediation by IR was much smaller in boys than in girls.

**Discussion:**

The direct association between adiposity and BP in healthy 4-year-old children is strong and IR plays an important mediating role. The strength of effects of IR and inflammation suggests sex differences in the complex interplay between BP, adiposity and inflammation.

## Introduction

High blood pressure (BP) in childhood and adolescence tracks into adult life and is an important risk factor for early cardiovascular disease [1]. The increasing incidence of hypertension in children has become a major concern in the context of the pandemic of overweight and obesity that occurred in the past few decades. Numerous reports have documented higher BP to parallel the rise in obesity in children and adolescents [2].

Obesity is a low-grade inflammatory state [3]. Adipose tissue is a major source of endocrine bioactive proinflammatory compounds, whereas the levels of anti-inflammatory adipokines such as adiponectin are reduced in obesity [4]. Moreover, resistance to insulin (IR), a hormone with anti-inflammatory action, is a hallmark of obesity-initiated metabolic syndrome, while inflammatory mediators additionally contribute to the IR state recognized in obesity [5].

A strong association between essential hypertension and inflammation has been demonstrated and significantly higher BP levels have also been found in subjects with highest IR indexes [6–8]. Studies in children have related high-sensitivity C-reactive protein (hsCRP), one of the most extensively studied inflammatory markers, to increased intimae-media thickness [9] and left ventricular hypertrophy [10] and cardiovascular risk [11].

In adults, sex differences have been described when associating inflammation and cardiovascular risk. Women have higher levels of hsCRP [12] and its association with BP tends to be stronger, which is only partly explained by different fat mass [13]. A recent study reported marked sexual dimorphism in the relationship of visceral and peripheral fat with BP variation in adolescence [14] but the differences in fat distribution are evident even earlier in childhood, with differences in total body fat starting before puberty [15, 16]. Actually, there is evidence that even before major hormonal changes occur during puberty, sex differences in hormonal levels might already exist and influence CV risk factors expression [17] but few studies explored these differences [18]. More evidence is needed to understand the operating mechanisms underlying the relations between overweight, IR, inflammation and BP in early childhood.

We hypothesized that low-grade inflammation and IR may play a role in the modulation of arterial BP during childhood, mediating at least part of the association between obesity and hypertension, and that these associations may differ between sexes. The aim of the present study was to assess the effect of indices of obesity on BP in 4-year-old boys and girls, and to which extent inflammation and IR mediate this association.

## Methods

### Study design and sample

The present study is based on the previously established cohort Generation XXI, a population-based birth cohort from northern Portugal (n = 8647) [19, 20]. At 4 years of the children’s age, the cohort was re-evaluated and 7458 (67.3%) children attended a face-to-face interview and physical examination at the study site, among whom 1524 (who had a cord blood sample stored) were invited to provide a fasting venous blood sample. From these we excluded: 18 children who had renal, cardiac or metabolic chronic diseases that were considered likely to interfere with BP, body composition or hsCRP values (no child had a history of usage of medication considered likely to interfere with the key study variables); 63 children with hsCRP values exceeding 10 mg/L, the accepted threshold for low-grade inflammation [21]; 159 children whose blood collection was not performed after an overnight fast of at least 8 hours; 22 children without valid BP measurements and 12 with only one isolated determination of BP were also excluded. The final sample for the current analysis included 1250 children, 609 girls and 641 boys.

When compared to the excluded children, those included in the final analysis had older mothers at the index child’s birth [mean (standard deviation (SD)): 29.3 (5.5) vs. 28.0 (6.1) years, p = 0.002] and were from families with a higher socioeconomic status (schooling of the parents >12 years: 29.5% vs. 19.9%, p = 0.002 and household income >1500€/month: 41.6% vs. 28.6%, p<0.001). The prevalence of preeclampsia during the index child’s gestation was lower in the included group (0.4% vs. 2.7%, p<0.001). No differences were found in the proportion of other gestational disorders, preterm delivery, presence of malformations at birth, sex of the child, birth weight or body mass index (BMI) at the 4 years old evaluation.

### Data collection and variables definition

The participants were evaluated at the study site, including face-to-face interviews of the caregivers and a physical examination of the children comprising anthropometric data measurements (namely height, weight and waist circumference) and BP evaluation.

Overweight and obesity of the parents was considered when at least one of the parents was affected; the World Health Organization criteria were considered for classification (overweight if BMI ≥ 25 kg/m2 and obesity if BMI ≥ 30 kg/m2), based on self-reported weight and height. If one of the child’s parents or siblings self-reported to have a previous diagnosis of hypertension or to be medicated with an anti-hypertensive drug at the moment of child’s evaluation, the child would be considered to have family history of hypertension. Gestational hypertensive disorders were defined by the presence of gestational hypertension or preeclampsia/eclampsia, as recorded on obstetrical records of the child’s mother during the index pregnancy and in the absence of a previous diagnosis of chronic hypertension. Classes of the sex-specific adequate birth weight for gestational age were defined according to the population-based Canadian reference curves (below the 10^th^ percentile, small for gestational age; equal to or above the 10^th^ percentile and below the 90^th^ percentile, adequate for gestational age; equal to or above the 90^th^ percentile, large for gestational age) [22].

At 4 years of age, body weight was determined to the nearest 0.1 kg in a digital scale (Seca) and height was determined in the upright position to the nearest 0.1 cm with a wall stadiometer (Seca). BMI was calculated as weight (in kg) divided by height (in m) squared. Waist circumference was measured at the umbilicus’ line to the nearest millimeter, with a tape measure. Waist circumference was indexed to height (WHtR in cm/m). BMI-for-age values were classified according to the World Health Organization growth reference data for BMI z-score for children below 5 years old, into the following categories: underweight (<-2 standard deviations (SD)); normal weight (-2SD to +1SD); at risk of overweight (+1SD to +2SD); overweight (>+2SD) and obesity (>+3SD) [23].

BP was evaluated with an aneroid sphygmomanometer (Erka Vario DeskModel) with an adequately sized cuff, by a trained examiner, twice with a 5-minute interval between measurements, with the subject in a seated position and the antecubital fossa supported at heart level, after at least a 5-minute rest. When the difference between the two determinations was larger than 5 mmHg for systolic (SBP) or diastolic BP (DBP) a third measurement was taken and the mean of the 2 closest values was considered. No significant differences were found between the average BP of the group with two measurements and the average of the 2^nd^ and 3^rd^ measurement in the group with three measurements (SBP: 106.8 vs. 106.7, p = 0.934; DBP: 73.5 vs. 74.0, p = 0.617 respectively). Systolic and diastolic BP were classified according to the American Academy of Pediatrics criteria and hypertension was considered as SBP and/or DBP equal or above to the 95^th^ percentile for sex, age and height [24].

Venous blood samples were obtained after 8 to 12 hours of overnight fast, for hsCRP, glucose and insulin measurement. All determinations were performed in the Clinical Pathology Department of Centro Hospitalar São João, Porto, Portugal. hsCRP was tested by immunonephelometric assays with CardioPhase hsCRP (Siemens Healthcare Diagnostics, Marburg, Germany), which holds a minimum threshold for detection of 0.16mg/L. The basal IR was assessed using the homeostasis model assessment of IR (HOMA-IR) [25] and logarithmized for the final analysis (log-HOMA-IR).

### Ethics Statement

The Generation XXI study and the present study protocol were approved by the Ethics Committee of Centro Hospitalar São João and Faculty of Medicine of the University of Porto and by the National Data Protection Commission. They comply with the Helsinki Declaration and the current national legislation. Written informed consent was obtained from caretakers on behalf of the children enrolled in our study and the children provided verbal assent to participate. The informed consent procedure was approved by the Ethics Committee.

### Statistical analysis

Associations between the continuous variables included in the Path models were quantified using Spearman correlation (ρ). The effect of the presence of obesity in the parents, family history of hypertension and birth weight classes for gestational age on SBP and DBP was evaluated using multiple linear regression models (adjusted for age (in months) and additionally for BMI z-score, when duly signed). Separate analysis was performed for boys and girls. Standard statistical analysis was performed using IBM SPSS Statistics for Windows, Version 20.0 (Armonk, NY).

Path Analysis, an extension of multivariate regression analysis which allows for the simultaneous estimation of interrelations between variables in a set, is being increasingly used to decompose and compare the magnitude of effects between variables with complex interrelations or to test the plausibility of mediation effects [26]. We conducted a Path Analysis assuming 2 possible causal models with hypothesized mechanisms linking obesity indices, either BMI z-score or WHtR, with SBP and DBP directly and the possible indirect effects mediated by hsCRP and log-HOMA-IR, adjusting for family history of hypertension (and additionally for age in months in the WHtR model). Models were fitted with Mplus software (Muthén and Muthén, Los Angeles, California); 95% confidence intervals were calculated by bootstrapping; and goodness of fit was evaluated using Qui-square test (degrees of freedom considered are indicated for each model), Confirmatory Fit Index (CFI; good fit ≥0.95; acceptable fit ≥0.90) and Root Mean Square Error of Approximation (RMSEA; good fit: <0.06; acceptable fit <0.08). For all models, we present effect estimates as non-standardized coefficients to facilitate the interpretation of the results. Dataset is provided as S1 Dataset.

## Results

Our sample included 1250 children with a mean (± SD) age of 53.0 ± 4.6 months, 51% males; their baseline characteristics are presented in Table 1. The overall prevalence of overweight and obesity was 6.2% and 2.6%, respectively, and 19.6% of children were at risk of overweight. Girls had a higher WHtR (49.6 vs. 49.0, p = 0.007) but BMI was not significantly different (16.2 vs. 16.0, p = 0.064). The prevalence of hypertension was 6.1% among normal weight children (5.0% in girls and 7.2% in boys) and 34.4% (46.7% in girls and 23.5% in boys) among obese children. Mean SBP was 97.5 ± 7.8 and 98.3 ± 8.3mmHg, and mean DBP was 56.4 ± 7.6 and 56.3 ± 7.9mmHg, in girls and boys, respectively.

**Table 1:** Characteristics of the study sample (n = 1250).

		Sex	
	All	Girls	Boys
	n = 1250	n = 609	n = 641
**Age (months), mean (SD)**	53.0 (4.6)	53.0 (4.5)	53.0 (4.7)
**Mother’s scholar years, mean (SD)**	10.7 (4.3)	10.6 (4.3)	10.7 (4.2)
**Monthly household income, n (%)**			
≤1500 €	712 (57.0)	350 (57.5)	362 (56.5)
>1500 €	538 (43.0)	259 (42.5)	279 (43.5)
**Overweight and obesity of the parents, n (%)**			
**Overweight**	431 (30.6)	210 (30.4)	221 (30.8)
**Obesity**	299 (21.2)	157 (22.8)	142 (19.8)
**Family history of hypertension, n (%)**	629 (51.8)	291 (49.7)	338 (53.8)
**Hypertensive gestational disorders** * **, n (%)**	27 (2.2)	13 (2.1)	14 (2.2)
**Birth weight for gestational age, n (%)** †			
Small (<10^th^ percentile)	173 (13.9)	91 (15.0)	82 (13.0)
Adequate (10^th^- 90^th^ percentile)	1024 (82.4)	490 (80.9)	534 (83.8)
Large (≥90^th^ percentile)	46 (3.7)	25 (4.1)	21 (3.3)
**BMI (kg/m^2^), mean (SD)**	16.1 (1.8)	16.2 (1.9)	16.0 (1.7)
**Waist-to-height ratio (cm/m), mean (SD)**	49.3 (3.5)	49.6 (3.8)	49.0 (3.3)
**Insulin resistance (HOMA-IR), median (IQR)**	0.71 (0.46–1.03)	0.74 (0.49–1.11)	0.66 (0.44–0.98)
**hsCRP (mg/L), median (IQR)**	0.4 (0.2–1.0)	0.5 (0.2–1.2)	0.3 (0.2–0.8)
**SBP (mmHg), mean (SD)**	97.9 (8.1)	97.5 (7.8)	98.3 (8.3)
**DBP (mmHg), mean (SD)**	56.4 (7.7)	56.4 (7.6)	56.3 (7.9)
**Hypertension, n (%)** ‡	120 (9.6)	56 (9.2)	64 (10.0)

SD, standard deviation; BMI, body mass index; HOMA-IR—homeostasis model assessment of insulin resistance; IQR, interquartile range; hsCRP, high sensitivity C-reactive protein; SBP, systolic blood pressure; DBP, diastolic blood pressure.

* Gestational hypertension and/or preeclampsia/eclampsia

^†^ According to Canadian fetal growth standard [22]

^‡^ Hypertension was defined as systolic and/or diastolic blood pressure equal or above to the 95th percentile for sex, age and height, according to American Academy of Pediatrics criteria and reference values [21].

The regression of child’s systolic and diastolic BP on obesity of the parents, family history of hypertension and child’s birth weight classes according to gestational age are shown in Table 2. The effect of these variables on BP after adjustment to child’s BMI was evaluated in order to decide if they should be included in the final Path analysis models. The presence of obesity in the parents had a significant effect only on girls’ SBP but this effect was no longer significant after adjustment for child’s BMI z-score. The existence of family history of hypertension also presented a positive effect on girl’s SBP and this effect persisted after adjustment for child’s BMI z-score. The class of birth weight according to the gestational age at birth was not significantly associated with BP, neither in girls nor in boys (Table 2). Considering these results, all the Path Models presented below were adjusted for family history of hypertension, a potential confounder of the association tested.

**Table 2:** Mean changes in systolic and diastolic blood pressure according to pre- and perinatal variables, independently of current child’s BMI z-score.

	Girls		Boys	
	Systolic BP	Diastolic BP	Systolic BP	Diastolic BP
	β (95%CI)	β (95%CI)	β (95%CI)	β (95%CI)
	p value	p value	p value	p value
**Obesity of the parents**				
**Model 1**	2.06 (0.62 to 3.50)	1.07 (-0.31 to 2.45)	1.39 (-0.14 to 2.92)	0.36 (-1.12 to 1.83)
	p = 0.005	p = 0.127	p = 0.075	p = 0.637
**Model 2**	0.71 (-0.62 to 2.05)	0.27 (-1.08 to 1.63)	0.83 (-0.62 to 2.28)	-0.10 (-1.52 to 1.33)
	p = 0.293	p = 0.691	p = 0.263	p = 0.894
**Family history of hypertension**				
**Model 1**	1.36 (0.13 to 2.60)	0.30 (-0.87 to 1.48)	0.92 (-0.32 to 2.16)	-0.09 (-1.28 to 1.10)
	p = 0.030	p = 0.613	p = 0.144	p = 0.887
**Model 2**	1.88 (0.77 to 2.99)	0.61 (-0.53 to 1.74)	1.09 (-0.07 to 2.26)	0.05 (-1.10 to 1.19)
	p = 0.001	p = 0.296	p = 0.066	p = 0.936
**Birth weight class**				
**Small for gestational age**				
**Model 1**	-1.65 (-3.33 to 0.03)	-0.90 (-2.51 to 0.71)	0.27 (-1.55 to 2.10)	-0.06 (-1.81 to 1.69)
	p = 0.054	p = 0.275	p = 0.770	p = 0.944
**Model 2**	-0.30 (-1.84 to 1.24)	-0.07 (-1.64 to 1.50)	0.88 (-0.85 to 2.60)	0.42 (-1.27 to 2.11)
	p = 0.699	p = 0.926	p = 0.318	p = 0.625
**Large for gestational age**				
**Model 1**	-0.09 (-3.19 to 3.01)	-1.94 (-4.92 to 1.03)	1.12 (-2.29 to 4.53)	-0.59 (-3.86 to 2.68)
	p = 0.955	p = 0.200	p = 0.519	p = 0.722
**Model 2**	-2.24 (-5.08 to 0.60)	-3.25 (-6.14 to -0.36)	0.04 (-3.18 to 3.25)	-1.46 (-4.61 to 1.69)
	p = 0.121	p = 0.027	p = 0.983	p = 0.364

The values presented are β regression coefficients and 95% confidence intervals (shown in parenthesis). Model 1 is adjusted to each variable in the table and additionally for age (in months). Model 2 is similar to Model 1 but additionally adjusted for child’s BMI z-score.

β, regression coefficient; 95%CI, 95% confidence interval; BP, blood pressure.

### BMI model

At 4 years of age, SBP increased significantly with BMI, by 3.2 mmHg and 2.6 mmHg per SD of BMI z-score (p<0.001), in girls and boys, respectively (1 SD BMI z-score is approximately 1 kg/m^2^ for girls and boys at this age). DBP also increased significantly with BMI z-score, by 1.9 mmHg and 2.0 mmHg per SD, in girls and boys (p<0.001) (Table 3).

**Table 3:** Magnitude of standardized direct and indirect effects of z-score BMI on SBP and DBP, calculated by path analysis.

	Girls				Boys			
	Systolic BP		Diastolic BP		Systolic BP		Diastolic BP	
	β (95%CI)	% of effect	β (95%CI)	% of effect	β (95%CI)	% of effect	β (95%CI)	% of effect
**Total BMI z-score**	3.17 (2.69 to 3.65)	-	1.86 (1.36 to 2.36)	-	2.62 (2.19 to 3.04)	-	2.01 (1.62 to 2.40)	-
	p<0.001		p<0.001		p<0.001		p<0.001	
**Direct**	2.68 (2.14 to 3.21)	84.5	1.52 (0.96 to 2.08)	81.7	2.44 (1.86 to 2.00)	93.1	1.92 (1.51 to 2.32)	95.5
	p<0.001		p<0.001		p<0.001		p<0.001	
**Indirect hsCRP**	0.10 (-0.02 to 0.22)	3.1	0.09 (-0.03 to 0.21)	4.8	0.01 (- 0.03 to 0.05)	0.4	- 0.01 (-0.05 to 0.03)	0.5
	p = 0.106		p = 0.133		p = 0.609		p = 0.509	
**Indirect log-HOMA-IR**	0.40 (0.19 to 0.60)	12.6	0.25 (0.02 to 0.48)	13.5	0.17 (0.02 to 0.31)	6.5	0.11 (-0.03 to 0.24)	5.5
	p<0.001		p = 0.033		p = 0.027		p = 0.129	
**Model fit tests**								
**Qui-square (d.f.)**	0.669 (2)		0.669 (2)		0.080 (2)		0.080 (2)	
	p = 0.716		p = 0.716		p = 0.961		p = 0.961	
**CFI**	1.000		1.000		1.000		1.000	
**RMSEA estimate**	<0.01		<0.01		<0.01		<0.01	

The values presented are β regression coefficients and 95% confidence intervals (shown in parenthesis) and the models are additionally adjusted for family history of hypertension.

β, regression coefficient; 95%CI, 95% confidence interval; BMI z-score, body mass index z-score; BP, blood pressure; log-HOMA-IR, logarithmized basal IR assessed by Homeostasis model assessment; hsCRP, high sensitivity C-reactive protein; d.f., degrees of freedom; CFI, Confirmatory Fit Index; RMSEA, Root Mean Square Error of Approximation.

In both sexes, SBP was positively correlated with log-HOMA-IR (ρ = 0.308, p<0.001 in girls and ρ = 0.193, p<0.001) and the same was verified for DBP (ρ = 0.181, p<0.001 in girls and ρ = 0.149, p<0.001 in boys). Both SBP and DBP were positively correlated with hsCRP levels but only in girls (SBP: ρ = 0.151, p<0.001 in girls and ρ = 0.031, p = 0.433 in boys; DBP: ρ = 0.164, p<0.001 in girls and ρ = -0.012, p = 0.760 in boys).

BMI z-score was significantly correlated with log-HOMA-IR in both sexes (ρ = 0.350, p<0.001 and ρ = 0.222, p<0.001, in girls and boys, respectively), whereas it was significantly correlated with hsCRP only in girls (ρ = 0.165, p<0.001 compared to ρ = 0.044, p = 0.235 in boys).

The goodness-of-fit of the BMI z-score models was tested and confirmed by CFI (1.000) and RMSEA (<0.01) and these values suggest a good global fitness (Table 3). When considering the path analysis model for BMI z-score on SBP, in both sexes, the association of adiposity and SBP was mainly explained by a significant direct effect, responsible for 84.5% and 93.2% of the association, in girls and boys, respectively. Nevertheless, in both sexes, a significant part of the effect of adiposity on SBP was indirectly mediated by IR, which explained 12.4% and 6.3% of the association, in girls and boys, respectively. In girls, the association of adiposity and DBP was mainly explained by a significant direct effect responsible for 81.7% of the association; however, a significant part of the association was indirectly mediated by IR (13.5%). In boys, the association of z-score BMI and DBP was uniquely explained by a strong direct effect, responsible for 95.4% of the total association (Table 3).

### WHtR model

SBP increased significantly with WHtR, by +0.68 per cm/m, in girls and in boys (p<0.001). DBP also increased significantly with WHtR, by +0.42 mmHg and +0.72 mmHg per cm/m, in girls and boys (p<0.001), respectively (Table 4).

**Table 4:** Magnitudes of standardized direct and indirect effects of WHtR on SBP and DBP, calculated by path analysis.

	Girls				Boys			
	Systolic BP		Diastolic BP		Systolic BP		Diastolic BP	
	β (95%CI)	% of effect	β (95%CI)	% of effect	β (95%CI)	% of effect	β (95%CI)	% of effect
**Total WHtR**	0.682(0.541 to 0.824)	-	0.423 (0.280 to 0.566)	-	0.682 (0.555 to 0.809)	-	0.717 (0.590 to 0.844)	-
	p<0.001		p<0.001		p<0.001		p<0.001	
**Direct**	0.505 (0.356 to 0.654)	74.1	0.308 (0.156 to 0.459)	72.8	0.615 (0.486 to 0.745)	90.2	0.687 (0.561 to 0.812)	95.8
	p<0.001		p<0.001		p<0.001		p<0.001	
**Indirect hsCRP**	0.037 (0.000 to 0.073)	5.4	0.031 (-0.002 to 0.064)	7.3	0.002 (-0.012 to 0.016)	0.3	-0.005 (-0.020 to 0.010)	0.7
	p = 0.052		p = 0.068		p = 0.778		p = 0.497	
**Indirect log-HOMA-IR**	0.140 (0.093 to 0.187)	20.5	0.085 (0.031 to 0.138)	20.1	0.064 (0.024 to 0.105)	9.4	0.036 (0.001 to 0.071)	5.0
	p<0.001		p = 0.002		p = 0.002		p = 0.046	
**Model fit tests**								
**Qui-square (d.f.)**	9.051 (4)		9.051 (4)		6.594 (4)		6.594 (4)	
	p = 0.060		p = 0.060		p = 0.159		p = 0.159	
**CFI**	0.981		0.972		0.986		0.983	
**RMSEA estimate**	0.044		0.044		0.030		0.030	

The values presented are β regression coefficients and 95% confidence intervals (shown in parenthesis) and the models are additionally adjusted for family history of hypertension and for age (in months).

β, regression coefficient; 95%CI, 95% confidence interval; WHtR, waist-to-height ratio; BP, blood pressure; log-HOMA-IR, logarithmized basal IR assessed by Homeostasis model assessment; hsCRP, high sensitivity C-reactive protein; d.f., degrees of freedom; CFI, Confirmatory Fit Index; RMSEA, Root Mean Square Error of Approximation.

WHtR significantly correlated with log-HOMA-IR in both sexes, with Spearman coefficients of 0.224 and 0.165 (both p<0.001), in girls and boys, respectively. As described above for BMI, WHtR significantly correlated with hsCRP only in girls, with a Spearman coefficient of 0.168 (p<0.001).

The goodness-of-fit of the WHtR models was tested and confirmed by CFI (values from 0.972 to 0.986) and RMSEA (values from 0.030 to 0.044) and these values suggest a good global fitness (Table 4). In boys, the direct effect of WHtR on SBP explained 90.2% of the total association and 9.4% of the association was mediated by log-HOMA-IR. In girls, we found that the association of WHtR with SBP was mainly explained by a significant direct effect (74.1%) and an indirect effect mediated by IR (20.5%), whereas the effect of hsCRP was only borderline significant (Table 4). For DBP, the same was found in girls, with the majority of the association being explained by the direct effect (72.8%) of WHtR and by an indirect effect mediated by IR (20.1%), and the effect of hsCRP mediating the association of adiposity on BP was not significant. The model for WHtR was additionally adjusted for age and family history of hypertension.

## Discussion

The results of our study confirm a strong association between obesity indices, both BMI and WHtR, and BP at 4 years of age. Our Path Analysis suggests that, regardless of the measure of obesity considered, most of the association of adiposity with SBP is apparently a direct effect in both sexes, explaining 74.1 to 93.2% of the total association. In addition, significant mediation by IR was observed particularly for WHtR. A similar pattern was observed for DBP, with most of the effect being direct, especially in boys, and some impact of IR particularly on WHtR and in girls (20% vs. 5% in boys). Mediation by inflammation did not reach statistical significance in either sex.

In our sample of 4-year-old children, the finding of a strong crude linear association between measures of obesity and both SBP and DBP is consistent with recent literature reporting a significant impact of obesity on the prevalence of childhood hypertension [2]. The magnitude of the association between obesity and BP is difficult to compare between studies, for methodological reasons. Still, our findings are well in line with previous studies of preschool children, with approximately 20% of hypertension prevalence in obese children [27, 28].

In this study, we used a novel biostatistical approach to determine the quantitative impact of physiopathological mechanisms established in adolescents and adults, i.e. low-grade inflammation and IR, on the development of obesity-related hypertension in early life. Path Analysis was applied to examine the comparative strength of direct and indirect associations between adiposity and BP. While the cross-sectional nature of our study requires careful interpretation with regard to cause-effect relationships, the results obtained by Path Analysis are consistent with previously reported results, with both SBP and DBP showing strong independent associations with both BMI z-score and WHtR, which were partially mediated by IR.

We decided to analyze both BMI z-score and WHtR since it is still controversial which adiposity measure is most closely correlated with BP and cardiovascular risk in children. In adults, central adiposity appears to be more strongly associated with adult cardiovascular disease and diabetes risk [29], and, in children, waist circumference was also suggested as being a better indicator of a range of cardiovascular risk factors, including BP [30]. Still, the vast majority of existing pediatric literature has used BMI to correlate BP and obesity, and a recent cohort study as well as a systematic review found no evidence for superior identification of children with increased cardiometabolic risk by use of waist circumference relative to BMI [31, 32]. The need for adjustment of waist circumference to height is equally controversial as studies in children yielded conflicting results [33, 34]. Adjustment for height appears to alter associations with BP; one study showed that when obesity measures were height-indexed only waist remained significantly positively associated with hypertension risk [34].

In this study, HOMA-IR was significantly correlated with SBP and DBP in both sexes, in keeping with previous findings in children [8]. Path Analysis suggested that the impact of adiposity on SBP is, to a significant part, indirectly mediated by IR in both sexes, but more markedly in girls. In accordance with previous observations, the effect attributable to IR was stronger with respect to WHtR than to BMI [8]. IR is not only the crucial disease mechanism underlying type 2 diabetes but also constitutes an important cardiovascular risk factor operative from childhood age. In the Bogalusa Heart study, significant positive correlations between fasting insulin and BP in children and adolescents were found and childhood IR was followed by higher BP at young adult age [35]. The authors examined the relationship between fasting insulin and glucose at baseline and longitudinal changes in BP, thus allowing the suggestion of a causation pattern, with IR as a possible determinant of BP levels in children [35].

Obesity is a state of glucose dysregulation, high levels of circulating insulin and reduced sensitivity to the metabolic actions of insulin, and some authors point that the higher BP values in obese individuals might result from persistent sympathetic overactivation and volume overload that are characteristic of IR, and that hyperinsulinemia might further increase sodium reabsorption, additionally contributing to additional volume overload independently of obesity [8].

Some studies in prepubertal children showed that girls are intrinsically more insulin resistant than boys [36] and that some differences between sexes in body composition and fat distribution can occur even before puberty [16]. Thus, the stronger effect of IR on BP seen in girls might be dependent on those differences but can also associate with other pathophysiological mechanisms not addressed in the current analysis, such as hyperleptinemia and resistance to leptin. In a recent study, a significant association between HOMA-IR and serum leptin was found, independently of adiposity levels [37] and it was hypothesized that serum leptin concentration might be an important predictor of IR. In children, leptin levels are known to differ between sexes, even before puberty, with marked rises in serum leptin concentrations in young girls [38]. Thus, leptin differences between sexes could account for some of the sex differences found in the current study.

In girls, both SBP and DBP correlated significantly with hsCRP levels but in the Path Analysis, inflammation did not contribute significantly to the association of BP and adiposity measures. The correlation found between hsCRP and BP level is in line with the results of previous studies focusing on the relation between obesity, low-grade inflammation and BP in healthy children. In 137 Bulgarian children aged 6 to 10 years, hsCRP concentrations increased with the degree of central obesity and were correlated with SBP even after controlling for adiposity [39]. Other studies in older children noted an association between metabolic syndrome components and hsCRP which however did not hold after adjustment for confounders [11, 40]. In a representative sample of 16,335 American children aged 1 to 19 years a consistent association between inflammation and obesity was demonstrated, which was significant from age 3 years onward and became much stronger with increasing age [41]. The authors hypothesized that inflammatory markers may not be immediately elevated with obesity onset in early childhood. Also in our sample, the correlation of hsCRP with BP was weak and of borderline statistical significance in multivariate models including measures of obesity, possibly implying that longer periods of exposure to excessive fat mass environment might be required for the intrinsic effect of inflammation on BP to become apparent. Brown et al. reinforced this hypothesis by comparing two cohorts of healthy children with a mean age of 5.5 and 8.5 years respectively [42]. In the younger group, hsCRP was not significantly related to either adiposity measures or BP, whereas highly significant correlations between hsCRP and adiposity measures as well as BP were evident in the older group which, as in our study, were attenuated when adjusted for covariates [41]. As the follow-up of our birth cohort is ongoing, we hope to contribute valuable longitudinal information to further unravel these intricate associations.

The mediation by hsCRP was not significant in either sex. Nonetheless, despite non-significant, the point estimate was higher in girls than in boys and further studies should try to address this difference. A previous longitudinal study in 118 children showed consistently higher serum IL-6 levels in girls at 15 years but not at 8 years of age [43]. In pubertal adolescents and adults, the sexual dimorphism of adipose tissue mass and inflammatory activity could be explained by differential effects of sex steroids on the regulation of the production and secretion of inflammatory substances [12]. Such overt endocrine effects obviously could not explain the gender differences in our sample of 4-year-old children. However, previous studies in children showed that sex differences in body composition and fat distribution [16] and in sex steroid levels exist before puberty, with girls presenting higher levels of estrogens than boys [17, 44]. Without measuring sexual hormones the application of these explanations require caution and no definite conclusions can be drawn. Another potential explanation for sex differences might be given by gender-related differences in the antenatal programming of nephron endowment and blood pressure [45]. It has been hypothesized that male fetuses grow more rapidly, with less relative placental growth, rendering them more vulnerable to the effects of maternal malnutrition on nephron development and more prone to developing essential hypertension independent of postnatal obesity- and inflammation-related mechanisms [46].

Our comprehensive assessment of different anthropometric measures of obesity, blood pressure and markers of inflammation and insulin resistance in conjunction with an advanced multivariate biostatistical approach allowed us to establish a consistent mechanistic network linking obesity to blood pressure in a large sample of young pre-pubertal children. Our findings support the usefulness of WHtR as an age- and sex-independent measure of central obesity in very young children [47]. The observed subtle gender differences observed in this prepubertal cohort should stimulate further research into sex-hormone independent sexually dimorphic cardiovascular phenotypes.

While BP measurements in this study were performed by the auscultation method, using aneroid devices as recommended by current guidelines [24], our analysis was limited in several ways by the cross-sectional study design: the lacking opportunity to confirm elevated BP values by repeat measurements may have caused some overestimation of the calculated prevalence of hypertension in the study cohort [48]. Moreover, even though BP measurements were undertaken in a research environment, the white coat effect cannot be ignored. Furthermore, the cross-sectional character of our analysis precludes firm causality inferences regarding the interplay of obesity, inflammation, IR and BP. We hope to prospectively overcome this limitation by continued follow-up of the children, ideally through adulthood, in order to achieve a better understanding of the long-term implications and consequences of excess weight in early childhood.

### Conclusion

In conclusion, our results provide further evidence that obesity is strongly associated with high BP early in life. We demonstrate an important role of IR in mediating the association between adiposity and BP. The strength of effects of IR and inflammation suggests a sexual dimorphism in the complex interplay between BP, adiposity and inflammation.

## Supporting Information

S1 Dataset Database including all variables used in statistical analyses here reported. (ZIP)

## References

[1] Chen X, Wang Y. Tracking of blood pressure from childhood to adulthood: a systematic review and meta-regression analysis. Circulation 2008; 117(25):3171–3180. 10.1161/CIRCULATIONAHA.107.730366 18559702 PMC3568631

[2] Flynn J. The changing face of pediatric hypertension in the era of the childhood obesity epidemic. Pediatr Nephrol 2013; 28(7):1059–66. 10.1007/s00467-012-2344-0 23138756

[3] Tam CS, Clement K, Baur LA, Tordjman J. Obesity and low-grade inflammation: a paediatric perspective. Obes Rev 2010; 11(2):118–126. 10.1111/j.1467-789X.2009.00674.x 19845868

[4] Cao H. Adipocytokines in obesity and metabolic disease. J Endocrinol 2014; 220(2):T47–59. 10.1530/JOE-13-0339 24403378 PMC3887367

[5] Dandona P, Aljada A, Chaudhuri A, Mohanty P, Garg R. Metabolic syndrome: a comprehensive perspective based on interactions between obesity, diabetes, and inflammation. Circulation 2005; 111(11):1448–1454. 15781756 10.1161/01.CIR.0000158483.13093.9D

[6] Montecucco F, Pende A, Quercioli A, Mach F. Inflammation in the pathophysiology of essential hypertension. J Nephrol 2011; 24(1):23–34. 20437401 10.5301/jn.2010.4729

[7] Syrenicz A, Garanty-Bogacka B, Syrenicz M, Gebala A, Dawid G, Walczak M. Relation of low-grade inflammation and endothelial activation to blood pressure in obese children and adolescents. Neuro Endocrinol Lett 2006; 27(4):459–64. 16891995

[8] Lurbe E, Torro I, Aguilar F, Alvarez J, Alcon J, Pascual JM, Redon J. Added impact of obesity and insulin resistance in nocturnal blood pressure elevation in children and adolescents. Hypertension 2008; 51(3):635–41. 10.1161/HYPERTENSIONAHA.107.099234 18195166

[9] Elkiran O, Yilmaz E, Koc M, Kamanli A, Ustundag B, Ilhan N. The association between intima media thickness, central obesity and diastolic blood pressure in obese and owerweight children: a cross-sectional school-based study. Int J Cardiol 2013; 165(3):528–32. 10.1016/j.ijcard.2011.09.080 22014414

[10] Assadi F. C-reactive protein and incident left ventricular hypertrophy in essential hypertension. Pediatr Cardiol 2007; 28(4):280–285. 17563829 10.1007/s00246-006-0173-2

[11] Soriano-Guillén L, Hernández-García B, Pita J, Domínguez-Garrido N, Del Río-Camacho G, Rovira A et al High-sensitivity C-reactive protein is a good marker of cardiovascular risk in obese children and adolescents. Eur J Endocrinol 2008; 159(1):R1–4. 10.1530/EJE-08-0212 18450770

[12] Lakoski SG, Cushman M, Criqui M, Rundek T, Blumenthal RS, D’Agostino RB, Herrington DM. Gender and C-reactive protein: data from the Multiethnic Study of Atherosclerosis (MESA) cohort. Am Hear J 2006; 152(3):593–8. 10.1016/j.ahj.2006.02.015 16923436

[13] Pruijm M, Vollenweider P, Mooser V, Paccaud F, Preisig M, Waeber G et al Inflammatory markers and blood pressure: sex differences and the effect of fat mass in the CoLaus Study. J Hum Hypertens 2013; 27(3):169–75. 10.1038/jhh.2012.12 22495106

[14] Pausova Z, Mahboubi A, Abrahamowicz M, Leonard GT, Perron M, Richer L et al Sex differences in the contributions of visceral and total body fat to blood pressure in adolescence. Hypertension 2012; 59(3):572–9. 10.1161/HYPERTENSIONAHA.111.180372 22291448

[15] Staiano AE, Broyles ST, Gupta AK, Katzmarzyk PT. Ethnic and sex differences in visceral, subcutaneous, and total body fat in children and adolescents. Obesity (Silver Spring) 2013; 21(6):1251–5. 23670982 10.1002/oby.20210 PMC3735659

[16] He Q, Horlick M, Thornton J, Wang J, Pierson RN, Heshka S, Gallagher D. Sex and race differences in fat distribution among Asian, African-American, and Caucasian prepubertal children. J Clin Endocrinol Metab 2002; 87(5):2164–70. 11994359 10.1210/jcem.87.5.8452

[17] Shi L, Remer T, Buyken AE, Hartmann MF, Hoffmann P, Wudy S a. Prepubertal urinary estrogen excretion and its relationship with pubertal timing. Am J Physiol Endocrinol Metab 2010; 299:E990–E997. 10.1152/ajpendo.00374.2010 20858752

[18] Barba G, Casullo C, Dello Russo M, Russo P, Nappo A, Lauria F, Siani A. Gender-related differences in the relationships between blood pressure, age, and body size in prepubertal children. Am J Hypertens 2008; 21(9):1007–10. 10.1038/ajh.2008.228 18617882

[19] Larsen PS, Kamper-Jørgensen M, Adamson A, Barros H, Bonde JP, Brescianini S et al Pregnancy and birth cohort resources in europe: a large opportunity for aetiological child health research. Paediatr Perinat Epidemiol 2013; 27(4):393–414. 10.1111/ppe.12060 23772942

[20] Alves E, Correia S, Barros H, Azevedo A. Prevalence of self-reported cardiovascular risk factors in Portuguese women: a survey after delivery. Int J Public Heal 2012; 57(5):837–47. 10.1007/s00038-012-0340-6 22314542

[21] Pearson TA, Mensah GA, Alexander RW, Anderson JL, Cannon RO, Criqui M et al Markers of inflammation and cardiovascular disease: application to clinical and public health practice: A statement for healthcare professionals from the Centers for Disease Control and Prevention and the American Heart Association. Circulation 2003; 107(3):499–511. 12551878 10.1161/01.cir.0000052939.59093.45

[22] Kramer MS, Platt RW, Wen SW, Joseph KS, Allen A, Abrahamowicz M et al A new and improved population-based Canadian reference for birth weight for gestational age. Pediatrics 2001; 108(2):E35 11483845 10.1542/peds.108.2.e35

[23] De Onis M, Lobstein T. Defining obesity risk status in the general childhood population: which cut-offs should we use? Int J Pediatr Obes 2010; 5(6):458–60. 10.3109/17477161003615583 20233144

[24] The fourth report on the diagnosis, evaluation, and treatment of high blood pressure in children and adolescents. Pediatrics 2004; 114(2 Suppl 4th Report):555–576. 15286277

[25] Matthews DR, Hosker JP, Rudenski AS, Naylor BA, Treacher DF, Turner RC. Homeostasis model assessment: insulin resistance and beta-cell function from fasting plasma glucose and insulin concentrations in man. Diabetologia 1985; 28(7):412–419. 3899825 10.1007/BF00280883

[26] Gamborg M, Andersen PK, Baker JL, Budtz-Jorgensen E, Jorgensen T, Jensen G, Sorensen TI. Life course path analysis of birth weight, childhood growth, and adult systolic blood pressure. Am J Epidemiol 2009; 169(10):1167–1178. 10.1093/aje/kwp047 19357327 PMC2732973

[27] Gopinath B, Baur LA, Garnett S, Pfund N, Burlutsky G, Mitchell P. Body mass index and waist circumference are associated with blood pressure in preschool-aged children. Ann Epidemiol 2011; 21(5):351–7. 10.1016/j.annepidem.2011.02.002 21458728

[28] He Q, Ding ZY, Fong DY, Karlberg J. Blood pressure is associated with body mass index in both normal and obese children. Hypertension 2000; 36(2):165–70. 10948072 10.1161/01.hyp.36.2.165

[29] Balkau B, Deanfield JE, Després J-P, Bassand J-P, Fox KAA, Smith SC et al International Day for the Evaluation of Abdominal Obesity (IDEA): a study of waist circumference, cardiovascular disease, and diabetes mellitus in 168,000 primary care patients in 63 countries. Circulation 2007; 116(17):1942–51. 17965405 10.1161/CIRCULATIONAHA.106.676379 PMC2475527

[30] Watts K, Bell LM, Byrne SM, Jones TW, Davis EA. Waist circumference predicts cardiovascular risk in young Australian children. J Paediatr Child Heal 2008; 44(12):709–15. 10.1111/j.1440-1754.2008.01411.x 19077071

[31] Falaschetti E, Hingorani AD, Jones A, Charakida M, Finer N, Whincup P et al Adiposity and cardiovascular risk factors in a large contemporary population of pre-pubertal children. Eur Heart J 2010; 31(24):3063–72. 10.1093/eurheartj/ehq355 20972265 PMC3001590

[32] Reilly JJ, Kelly J, Wilson DC. Accuracy of simple clinical and epidemiological definitions of childhood obesity: systematic review and evidence appraisal. Obes Rev 2010; 11(9):645–55. 10.1111/j.1467-789X.2009.00709.x 20059704

[33] Chiolero A, Paradis G, Maximova K, Burnier M, Bovet P. No use for waist-for-height ratio in addition to body mass index to identify children with elevated blood pressure. Blood Press 2013; 22(1):17–20. 10.3109/08037051.2012.701376 22817261

[34] Willig AL, Casazza K, Dulin-Keita A, Franklin FA, Amaya M, Fernandez JR. Adjusting adiposity and body weight measurements for height alters the relationship with blood pressure in children. Am J Hypertens 2010; 23(8):904–10. 10.1038/ajh.2010.82 20414190 PMC2998040

[35] Jiang X, Srinivasan SR, Bao W, Berenson GS. Association of fasting insulin with longitudinal changes in blood pressure in children and adolescents. The Bogalusa Heart Study. Am J Hypertens 1993; 6(7 Pt 1):564–9. 8397996 10.1093/ajh/6.7.564

[36] Murphy MJ, Metcalf BS, Voss LD, Jeffery AN, Kirkby J, Mallam KM, Wilkin TJ. Girls at five are intrinsically more insulin resistant than boys: The Programming Hypotheses Revisited—The EarlyBird Study (EarlyBird 6). Pediatrics 2004; 113(1 Pt 1):82–6. 14702453 10.1542/peds.113.1.82

[37] Zuo H, Shi Z, Yuan B, Dai Y, Wu G, Hussain A. Association between serum leptin concentrations and insulin resistance: a population-based study from China. PLoS One 2013; 8(1):e54615 10.1371/journal.pone.0054615 23349940 PMC3551759

[38] Kaplowitz PB. Link between body fat and the timing of puberty. Pediatrics 2008; 121 Suppl:S208–17. 18245513 10.1542/peds.2007-1813F

[39] Galcheva S V, Iotova VM, Yotov YT, Bernasconi S, Street ME. Circulating proinflammatory peptides related to abdominal adiposity and cardiometabolic risk factors in healthy prepubertal children. Eur J Endocrinol 2011; 164(4):553–8. 10.1530/EJE-10-1124 21224406

[40] Valle Jiménez M, Estepa RM, Camacho RMM, Estrada RC, Luna FG, Guitarte FB. Endothelial dysfunction is related to insulin resistance and inflammatory biomarker levels in obese prepubertal children. Eur J Endocrinol 2007; 156(4):497–502. 17389466 10.1530/EJE-06-0662

[41] Skinner AC, Steiner MJ, Henderson FW, Perrin EM. Multiple markers of inflammation and weight status: cross-sectional analyses throughout childhood. Pediatrics 2010; 125(4):e801–9. 10.1542/peds.2009-2182 20194272 PMC2909480

[42] Brown DE, Mautz WJ, Warrington M, Allen L, Tefft HAT, Gotshalk L, Katzmarzyk PT. Relation between C-reactive protein levels and body composition in a multiethnic sample of school children in Hawaii. Am J Hum Biol 2010; 22(5):675–9. 10.1002/ajhb.21064 20737615

[43] Tam CS, Garnett SP, Cowell CT, Heilbronn LK, Lee JW, Wong M, Baur LA. IL-6, IL-8 and IL-10 levels in healthy weight and overweight children. Horm Res Paediatr 2010; 73(2):128–34. 10.1159/000277632 20190550

[44] Courant F, Aksglaede L, Antignac J-PP, Monteau F, Sorensen K, Andersson A-MM et al Assessment of circulating sex steroid levels in prepubertal and pubertal boys and girls by a novel ultrasensitive gas chromatography-tandem mass spectrometry method. J Clin Endocrinol Metab 2010; 95(1):82–92. 10.1210/jc.2009-1140 19933393

[45] Feig DI, Nakagawa T, Karumanchi SA, Oliver WJ, Kang D-H, Finch J, Johnson RJ. Hypothesis: Uric acid, nephron number, and the pathogenesis of essential hypertension. Kidney Int 2004; 66(1):281–7. 15200435 10.1111/j.1523-1755.2004.00729.x

[46] Eriksson JG, Kajantie E, Osmond C, Thornburg K, Barker DJP. Boys live dangerously in the womb. Am J Hum Biol 2010; 22(3):330–5. 10.1002/ajhb.20995 19844898 PMC3923652

[47] Taylor RW, Williams SM, Grant AM, Taylor BJ, Goulding A. Predictive ability of waist-to-height in relation to adiposity in children is not improved with age and sex-specific values. Obes (Silver Spring) 2011; 19(5):1062–8. 10.1038/oby.2010.217 20885392

[48] Steinthorsdottir SD, Eliasdottir SB, Indridason OS, Agustsdottir IM, Palsson R, Edvardsson VO. Prevalence of hypertension in 9- to 10-year-old Icelandic school children. J Clin Hypertens 2011; 13(10):774–9. 10.1111/j.1751-7176.2011.00496.x PMC8108873 21974766

